# Mild Cognitive Impairment Patients Have Higher Regulatory T-Cell Proportions Compared With Alzheimer's Disease-Related Dementia Patients

**DOI:** 10.3389/fnagi.2020.624304

**Published:** 2021-01-22

**Authors:** Jiping Fu, Jinhai Duan, Jianwei Mo, Hao Xiao, Yuedong Huang, Weiping Chen, Shaotong Xiang, Fan Yang, Yongjun Chen, Shuwen Xu

**Affiliations:** ^1^Eastern Department of Neurology, Guangdong Geriatrics Institute, Guangdong Provincial People's Hospital, Guangdong Academy of Medical Sciences, Guangzhou, China; ^2^Department of Neurology, First Affiliated Hospital of Shantou University Medical College, Shantou, China

**Keywords:** Alzheimer's disease, neuroinflammation, regulatory T-cells, immunosuppression, cytokines

## Abstract

**Objectives:** The role of neuroinflammation in the pathogenesis of Alzheimer's disease (AD) has attracted much attention recently. Regulatory T-cells (Tregs) play an important role in modulating inflammation. We aimed to explore the Treg-related immunosuppression status at different stages of AD.

**Methods:** Thirty healthy control (HC) subjects, 26 patients with mild cognitive impairment (MCI), 30 patients with mild probable AD-related dementia, and 28 patients with moderate-to-severe probable AD-related dementia underwent detailed clinical history taking, structural MRI scanning, and neuropsychological assessment. Peripheral blood samples were taken to measure the percentage of CD4^+^CD25^+^CD127^low/−^ Tregs by flow cytometry and the levels of interleukin (IL-10), interleukin (IL-35), and transforming growth factor β (TGF-β) by ELISA.

**Results:** The percentage of Tregs in the blood of MCI patients was the highest (9.24%); there was a significant difference between patients with MCI and patients with probable AD-related dementia. The level of TGF-β in patients with MCI (47.02 ng/ml) was significantly increased compared with patients with AD-related dementia. There were positive correlations between Treg percentage, IL-35, and Mini-mental state evaluation scores in patients with MCI and probable AD-related dementia.

**Conclusions:** Patients with MCI have stronger Treg-related immunosuppression status compared with patients with probable AD-related dementia.

## Introduction

Alzheimer's disease (AD), the most common cause of dementia in the elderly, is a global health concern with a huge burden for individuals and society. However, the pathogenesis of AD remains unknown. The failure of several drug clinical trials targeting beta amyloid (Aβ) in recent years has led to the question of the most widely known amyloid hypothesis (Panza et al., 2019). In addition to senile plaques and neurofibrillary tangles, chronic neuroinflammation is the third major pathological feature of AD (Kinney et al., 2018), and inflammatory responses have been observed in AD autopsy studies (Gomez-Nicola and Boche, 2015; Knezevic and Mizrahi, 2018). One study showed that the number of naive T-cells in patients with AD were reduced, while the effector memory T-cells were increased (Larbi et al., 2009). The innate immune cells, such as natural killer cells and neutrophils, in patients with mild cognitive impairment (MCI) were upregulated compared with those of patients with mild AD-related dementia (Le Page et al., 2015). Recent studies have shown that the most important component of senile plaque-Aβ has antimicrobial properties (Moir et al., 2018), suggesting that patients with AD may be immune to unidentified substances derived from chronic viral, bacterial, or fungal infections. The immune changes associated with the development and progression of AD have already been the hotspot in the field of AD research.

Immune regulation is the key to maintaining a stable internal environment of the human body. The human body produces rapid and specific responses through the strict regulation of the immune system once a pathogen attacks. In the regressive phase, these responses need to be inhibited to prevent chronic inflammation and tissue damage. Immunoregulatory cells—regulatory T-cells (Tregs) [cell phenotype can be defined as CD4^+^CD25^+^Foxp3^+^, Tregs hardly express CD127, so they can also be expressed by CD4^+^CD25^+^CD127^low/−^ (Seddiki et al., 2006)], participate in this important regulatory mechanism through the secretion of anti-inflammatory cytokines, such as interleukin-10 (IL-10), interleukin-35 (IL-35), transforming growth factor beta (TGF-β), and cell-cell contact, to prevent the occurrence of chronic inflammation and autoimmunity (Campbell, 2015). In amyotrophic lateral sclerosis (ALS), Tregs have protective effects that can delay the disease progression, and early reduction of Foxp3 levels can be used to screen patients with rapid progression (Zhao et al., 2012; Henkel et al., 2013). The accumulation of Tregs in cancer patients, such as breast cancer and liver cancer, was associated with poor prognosis. In addition to immunomodulatory effects, Tregs play a role in maintaining homeostasis and repairing damage in non-lymphoid organs (Spitz et al., 2016; Ito et al., 2019).

There is an immune imbalance in patients with AD, and microglia and astrocytes release cytotoxic substances, such as pro-inflammatory cytokines and oxygen free radicals, to cause neuronal damage and death (Hardy and Selkoe, 2002). Exploring the inhibitory inflammatory response in patients with AD is of great significance for understanding the pathogenesis of AD. There are few reports on the role of Tregs in AD and the findings are inconsistent (Rosenkranz et al., 2007; Saresella et al., 2010; Le Page et al., 2017). One of the studies found that the suppressive activity of Tregs was increased in patients with AD (Rosenkranz et al., 2007), while the other two studies found that the frequencies of Tregs were increased in patients with MCI. In the AD-related dementia stage, Tregs were reduced instead (Saresella et al., 2010; Le Page et al., 2017). Two animal experiments have different conclusions about the role of Tregs in AD due to the different stages of the AD mice models (Baruch et al., 2015; Dansokho et al., 2016). IL-35 is a newly discovered pure immunosuppressive cytokine mainly produced by Tregs, which plays an important role in various autoimmune diseases (Su et al., 2018). However, the expression status of IL-35 in AD has yet to be elucidated clearly. Since inflammation in patients with AD is a chronic progressive process, the effects of Tregs in different stages may be different. The aim of this study was to measure the levels of Tregs and their related cytokines (IL10, IL35, and TGF-β) in the peripheral blood of patients with probable AD and to understand the Treg-related immunosuppression status at different stages of AD.

## Materials and Methods

### Subjects

Patients with MCI and probable AD-related dementia were clinically diagnosed according to the 2011 National Institute on Aging-Alzheimer's Association (NIA-AA) workgroups on diagnostic guidelines for AD. The clinical dementia rating (CDR) scale was used to differentiate the severity of AD-related dementia (mild dementia: CDR = 1 point, moderate-to-severe dementia: CDR ≥ 2 points). Subjects who had a history of trauma, cerebrovascular disease, intracranial infection, mental disorder, and autoimmune disease were excluded. Each subject underwent clinical evaluation, neuropsychological assessment, laboratory examination, and head structural MRI. Mini-mental state evaluation (MMSE), Montreal cognitive assessment (MoCA), Hamilton anxiety scale (HAMA), Hamilton depression scale (HAMD), The Bayer Activities of Daily Living Scale (B-ADL), and Hachinski ischemia scale (HIS) were performed, except for patients with very severe AD-related dementia because they could not complete full scale assessment. The study enrolled 26 subjects with MCI, 30 patients with mild AD-related dementia, and 28 patients with moderate-to-severe AD from October 2017 to September 2018 in outpatient and inpatient departments of Guangdong Provincial People's Hospital. Another 30 healthy control (HC) subjects were selected as the control group. The research protocol was approved by Guangdong Provincial People's Hospital. All subjects or their guardians were informed of the content and the purpose of the study. All of them volunteered to participate in the study and signed written informed consent. Additional details of the clinical data of patients are summarized in Table 1.

**Table 1:** Patients' clinical data, percentage of Tregs, and related cytokine levels of four groups.

**Parameters**	**HC (*n =* 30)**	**MCI (*n =* 26)**	**Mild AD (*n =* 30)**	**Mod&severe AD (*n =* 28)**	**X^**2**^/F/H**	***P-*value**
Sex (M/F)	16/14	16/10	18/12	15/13	0.628	0.890
Age (years)	73.03 ± 9.21	75.19 ± 11.45	79.23 ± 10.54	79.39 ± 17.13	1.864	0.140
Education (years)	13.43 ± 3.79	12.85 ± 3.02	10.50 ± 5.19	11.21 ± 3.92	7.227	0.065
MMSE (score)	28.63 ± 1.35	25.46 ± 2.79	20.07 ± 3.36	11.54 ± 6.68	92.933	0.000
Tregs (%)	8.19 ± 1.97	9.24 ± 2.38	7.78 ± 1.26	7.42 ± 1.61	11.645	0.009
IL10 (pg/ml)	30.51 ± 17.06	32.89 ± 22.66	28.01 ± 12.31	32.38 ± 16.72	0.721	0.868
TGF-β (ng/ml)	37.92 ± 12.03	47.02 ± 16.28	38.31 ± 10.49	34.83 ± 7.87	9.885	0.020
IL35 (pg/ml)	21.17 ± 18.72	24.75 ± 26.33	19.95 ± 13.07	15.75 ± 12.12	1.386	0.709

*HC, healthy control; MCI, mild cognitive impairment; AD, Alzheimer's disease; Mod&severe, moderate and severe; MMSE, Mini-mental state evaluation; Tregs, regulatory T-cells; IL-10, interleukin-10; TGF-β, transforming growth factor β; IL-35, interleukin-35*.

### Tregs Analysis by Flow Cytometry

Fluorochrome-labeled monoclonal antibodies mainly CD4-FITC, CD127-PE, and CD25-APC (eBioscience, San Diego, USA), which identified Tregs, were added to flow measuring tubes. Fifty microliters of whole blood samples treated with EDTA-K3 were added to the tube, mixed well, and incubated for 20 min at room temperature in the dark. Then, the samples were added with 2 ml RBC lysing buffer and mixed well to lyse RBC, incubated for 10 min at room temperature in the dark, and centrifuged, after which the supernatant was discarded. Later, they were washed with 2 ml phosphate buffered saline containing 0.5% bovine serum albumin and centrifuged at 1,000 rpm for 5 min, after which the supernatant was discarded. The cells were resuspended in 300 μl fixative solution and placed at 4°C in the dark. Data were acquired by flow cytometry; fixed cells were analyzed using CellQuest/Diva software within 24 h; and the percentage of CD4^+^CD25^+^CD127^lo/−^ cells was obtained in CD4^+^ lymphocytes.

### IL-10, IL-35, and TGF-β Quantification

Sera were collected following the centrifugation of non-anticoagulated whole blood (1,000 × *g*, 20 min). Samples were frozen at −80°C until the day of analysis. The concentrations of IL-10, IL-35, and TGF-β were determined by ELISA technology. All experimental procedures were carried out in accordance with the instructions of the ELIAS kit (IL-35: XpressBio, Frederick, MD, USA; IL-10 and TGF-β: 4A Biotech Co, Beijing, China). A standard curve was drawn based on the concentrations of the standard solution and their OD_450_ value. The concentrations of IL-10, IL-35, and TGF-β in the sample can be calculated from the standard curve according to their OD values.

### Statistical Analysis

Data were processed using Statistical Package for the Social Sciences (SPSS) 25 software. For count data, the chi-square test was conducted. For normally distributed data, one-way ANOVA was conducted. A comparison involving non-normally distributed variables was conducted by the Kruskal–Wallis H test. For significant Kruskal–Wallis tests, pairwise comparisons were completed using the Mann–Whitney *U*-test with Bonferroni correction. The relationship between Tregs, IL-10, IL-35, TGF-β, and cognitive function was calculated by Spearman's correlation analysis after controlling for age. Only patients with MCI and AD were included in the correlation analyses.

## Results

### The Percentage of Tregs and Concentrations of IL-10, IL-35, and TGF-β Comparison Among Four Groups

**Figure 1:**
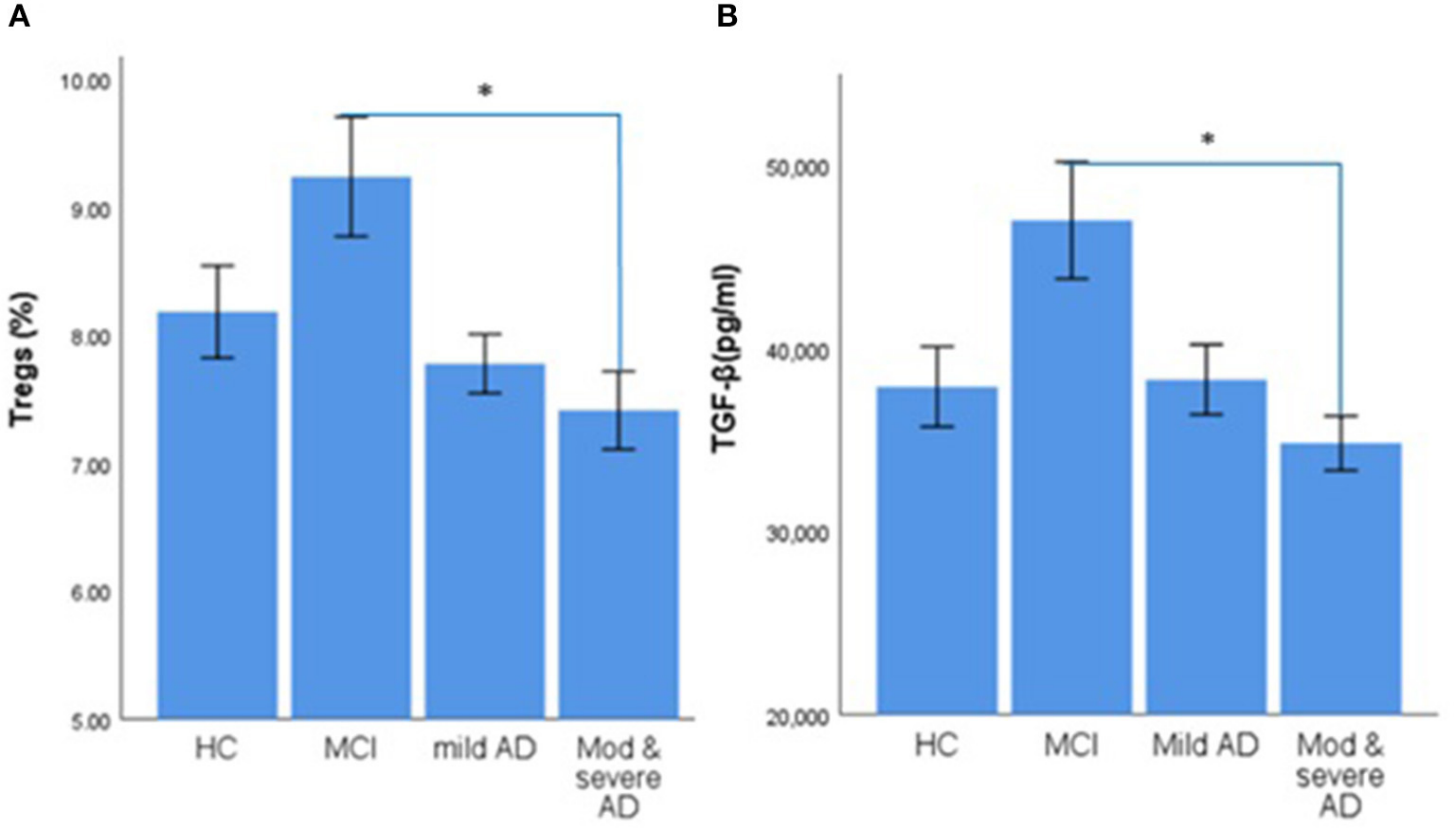
Error bar charts show the difference in mean ± standard error of the mean (SEM) values of regulatory T cells (Tregs) and transforming growth factor β (TGF-β) in peripheral blood of healthy control (HC), mild cognitive impairment (MCI), mild Alzheimer's Disease (AD) dementia, and moderate-to-severe AD dementia subjects. **(A)** Percentage of CD^4+^CD^25+^CDl27^low/−^ Tregs. **(B)** The Concentration of TGF-β. The asterisks (^*^) means *p* < 0.0083.

1. The percentage of Tregs in the blood of patients with MCI was the highest (9.24%), followed by HC subjects (8.19%), and the proportion of Tregs in patients with moderate-to-severe AD-related dementia was the lowest (7.42%). There was a significant difference in the percentage of Tregs between patients with MCI and patients with moderate-to-severe AD-related dementia (the value of *p* was 0.004; Table 1 and Figure 1).
2. The level of TGF-β in patients with MCI (47.02 ng/ml) was the highest and significantly increased compared with patients with moderate-to-severe AD-related dementia (the value of *p* was 0.002), the group that had the lowest level of TGF-β (34.83 ng/ml; Table 1 and Figure 1).
3. There were no significant differences in the levels of IL-10 and IL-35 among the four groups (Table 1).
4. The percentage of Tregs and the level of TGF-β in patients with MCI were significantly increased compared with patients with AD-related dementia, which were composed of patients with mild and moderate-to-severe AD-related dementia (for Tregs, the value of *p* was 0.002; for TGF-β, the value of *p* was 0.003).

### Correlation Between Tregs, IL-10, IL-35, TGF-β, and Cognitive Function in Patients With MCI and AD-Related Dementia

**Figure 2:**
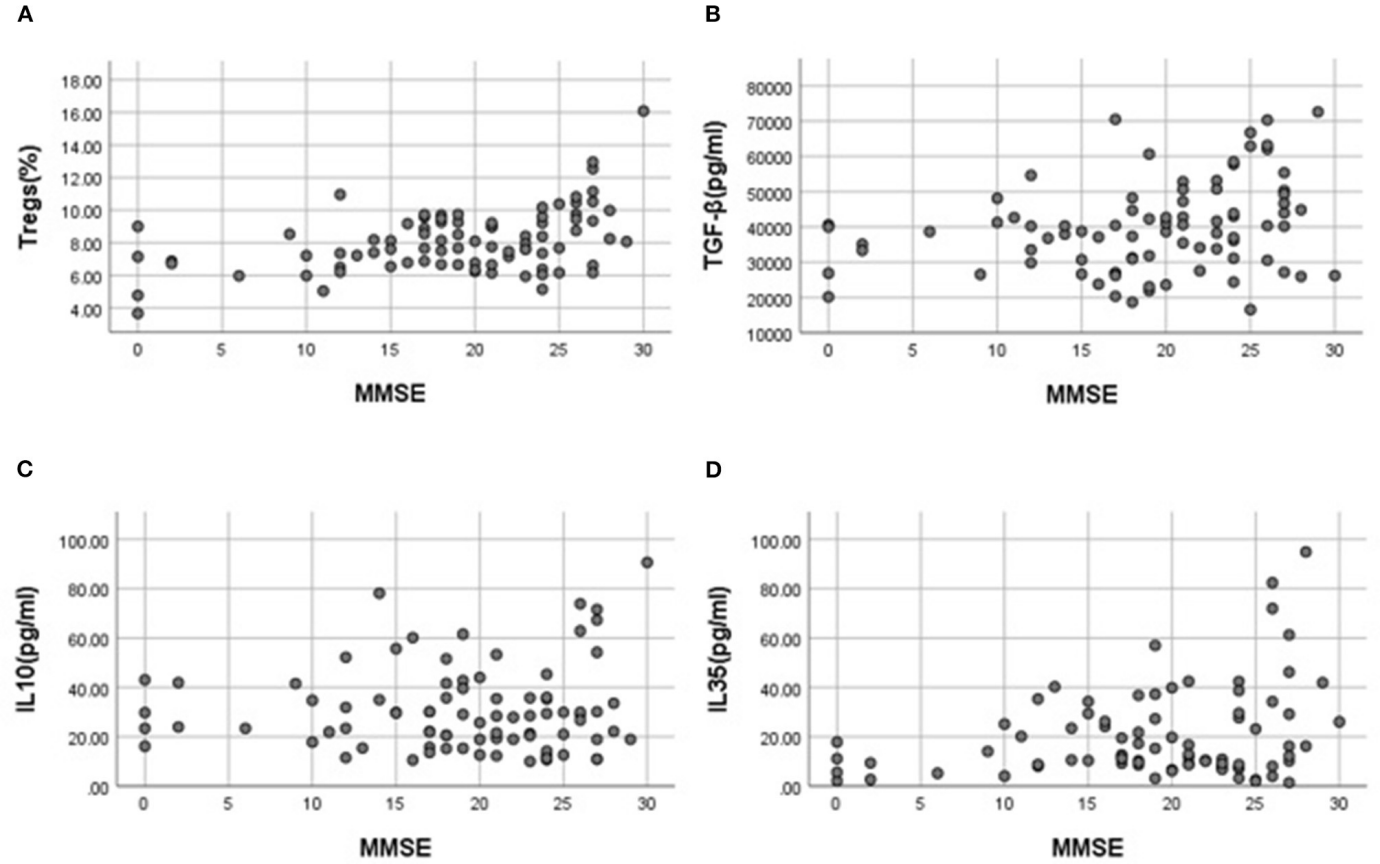
Scatter plots show correlation between regulatory T cells (Tregs), transforming growth factor β (TGF-β), interleukin 10 (IL10), interleukin 35 (IL35), and cognitive function after controlling for age in MCI and AD dementia patients. **(A)** Correlation between percentage of CD^4+^CD^25+^CDl27^low/−^ Tregs and Mini-mental state evaluation (MMSE) scores (the partial correlation coefficient was 0.445, *P-value* was 0.001). **(B)** Correlation between concentration of TGF-β and MMSE scores (*P* > 0.05). **(C)** Correlation between concentration of IL10 and MMSE scores (*P* > 0.05). **(D)** Correlation between concentration of IL35 and MMSE scores (the partial correlation coefficient was 0.285, *P-value* was 0.009).

There was a positive correlation between the percentage with Tregs and the scores of MMSE (the partial correlation coefficient was 0.445, the value of *p* was < 0.001). A mild, positive significant correlation was found between MMSE scores and TGF-β (the partial correlation coefficient was 0.292, the value of *p* was 0.007), MMSE scores, and IL-35 (the partial correlation coefficient was 0.285, the value of *p* was 0.009). There was no correlation between IL-10 and MMSE scores (Figure 2).

## Discussion and Conclusion

The immune system plays a key role in the pathogenesis of AD. Autopsy found that activated microglia clustered around amyloid plaques (Calsolaro and Edison, 2016). Many AD-risk genes, including CR1, CD33, and TREM2, are related to the immune system (Jones et al., 2010; Hollingworth et al., 2011). Clinical studies using PET ligands that bind to activated microglia provide further *in vivo* evidence for understanding the role of neuroinflammation in patients with AD (Hamelin et al., 2016). Whether or when neuroinflammation is protective, harmful, or both may depend on the stage of the disease and the genetic subtype. The lymphoid system in the brain can drain the central nervous system (CNS)-derived antigen and produce acquired immunity against CNS. The CNS-derived antigen is mainly drained through adjacent structures such as choroid plexus (CP), pia mater space, and deep cervical lymph nodes. Dendritic cells, T cells infiltrate into the brain through the same pathway (Korn and Kallies, 2017).

Our study found that patients with MCI had the highest proportion of Tregs, followed by HC subjects, and patients with moderate-to-severe probable AD-related dementia had the lowest proportion of Tregs. Among the inhibitory cytokines secreted by Tregs, patients with MCI had the highest level of TGF-β, which was statistically significant compared with patients with AD-related dementia. The data above indicated that patients with MCI had enhanced the immunosuppressive function, which was consistent with two other studies (Saresella et al., 2010; Le Page et al., 2017). Saresella et al. (2010) found that the subpopulation of Tregs with the most powerful suppressive ability, PD1^−^CD4^+^CD25^+^Foxp3^+^ Tregs, was significantly increased in patients with MCI, and Treg-mediated immunosuppression was stronger compared to that of HC and patients with AD-related dementia. Other studies reported that the early stage of patients with AD had an enhanced pro-inflammatory response (Heneka et al., 2015; Yasuno et al., 2017; Oberstein et al., 2018). Patients with MCI had an increased proportion of Th17 (Oberstein et al., 2018). The proportion of peripheral cytotoxic T-cells had increased even in the preclinical stage of AD with no cognitive changes but with only the deposition of Aβ (Yasuno et al., 2017). Combined with the similar study published in 2017 (Le Page et al., 2017), we could speculate that the proportion of Tregs and TGF-β levels in patients with MCI may be a compensatory increase to reduce the excessive immune response. In AD-related dementia stage, especially in moderate-to-severe AD-related dementia stage, the compensatory mechanism disappears, the protective effect of Tregs and TGF-β disappears. Larbi et al. (2009) found that the distribution of CD4^+^CD25^+^Tregs gradually shifted to memory subpopulation with age, and the proportion of all Treg subgroups in patients with AD-related dementia decreased compared with healthy elderly patients. Recently, a study also found that the percentage of total Tregs and resting Tregs significantly decreased in the middle stage of patients with AD, as compared to HC subjects, suggesting an impairment of the immune reserve in patients with AD-related dementia (Ciccocioppo et al., 2019). The adaptive immune system of patients with AD-related dementia was affected by continuous antigenic stimulation, which may result in premature immunosenescence. In our study, the proportion of Tregs, as well as Treg-related cytokines, IL-35, and TGF-β, positively correlated with the MMSE score. The worse the cognitive function, the lower was the proportion of Tregs and Treg-related cytokines. The premature immunosenescence may explain why the proportion of Tregs in patients with moderate-to-severe AD-related dementia decreased in the study.

The above studies indicated that Tregs might be involved in the pathogenesis of AD, but it is not clear through which mechanism Tregs can affect the pathological process of AD. Baruch et al. (2015) found that the transient depletion of Tregs increased the production of IFN-γ at the CP in 5XFAD mice and upregulated the expression of leukocyte trafficking molecules such as ICAM-1, CXCL10, and CCL2 by the CP, followed by an increased accumulation of mononuclear-macrophages at cerebral site of plaque formation. Cerebral Aβ plaque burden in the brain was reduced and cognitive decline was reversed. On the contrary, an increased number of peripheral Tregs in 5XFAD mice was associated with higher burden of cerebral Aβ plaque and worse cognitive function.

However, some animal studies drew the opposite conclusion. Dansokho et al. (2016) found that transient clearance of Tregs significantly reduced the aggregation of activated microglia to Aβ and accelerated the cognitive impairment of mice but did not change the deposition of Aβ, while the expansion of Tregs increased the number of plaque-associated microglial cells and improved the cognitive function. Further analysis suggested that Tregs may contribute to promote a type I IFN-dependent beneficial activation profile of microglial in response to amyloid deposition (Dansokho et al., 2016).

The role of Tregs in AD animal models is inconsistent across different studies, probably due to the modulation of Tregs in AD mice models at different disease stages. Baruch et al. (2015) interfered peripheral Tregs in 5XFAD mice at 4–5 months old after significant pathological changes, such as the deposition of Aβ and gliosis, have developed, whereas Dansokho et al. (2016) modulated Tregs in APPPS1 mice at 5–6 weeks of age when the deposition of Aβ and gliosis has just emerged. At early disease stages, Tregs may promote type I IFN-dependent beneficial activation profile in microglia. At later disease stages, the accumulation of brain-derived signals associated with disease progression may lead to different responsiveness of CP to Tregs, resulting in Treg-mediated alteration of CP and impaired recruitment of inflammation-resolving leukocytes to CNS. It is still necessary to do more studies to explore the changes of Tregs in different stages of AD and how the changes affect the course of AD.

There were several limitations in this study: (1) The authors did not follow-up the changes of cognition and the frequency of Tregs and its related cytokines in enrolled subjects. The relationship between Tregs and the progression of the disease could not be clarified. (2) The sample sizes were small and the statistical power of the study was low. (3) The subjects did not get a spinal tap, so the number of Tregs in cerebrospinal fluid (CSF) could not be detected, and the immune suppression status of patients with AD could not be accurately reflected. (4) We did not include any CSF biomarkers or PET imaging that some patients with MCI or dementia with other etiologies may be enrolled in; this could lead to confounding result. A study published in 2018 detected the Aβ and tau protein in patients with MCI, and found that there was no difference between HC and MCI due to AD (Oberstein et al., 2018). Therefore, it is necessary to expand the sample sizes to further explore the changes in the number and function of Tregs in the peripheral blood and CSF of patients with AD.

## Data Availability Statement

The raw data supporting the conclusions of this article will be made available by the authors, without undue reservation.

## Ethics Statement

The studies involving human participants were reviewed and approved by Guangdong Provincial People's Hospital. The patients/participants provided their written informed consent to participate in this study. Written informed consent was obtained from the individual(s) for the publication of any potentially identifiable images or data included in this article.

## Author Contributions

SXu and JD: designed and conceptualized the study. JF: analyzed the data and drafted the manuscript for the intellectual content. JM, HX, YH, and WC: a major role in the acquisition of clinical data and implementation of neuropsychological scale assessment. FY, SXi, and YC: detected the level of Tregs, IL-10, IL-35, and TGF-β in the peripheral blood. All authors contributed to the article and approved the submitted version.

## Conflict of Interest

The authors declare that the research was conducted in the absence of any commercial or financial relationships that could be construed as a potential conflict of interest.
